# Uncoupling fork speed and origin activity to identify the primary cause of replicative stress phenotypes

**DOI:** 10.1074/jbc.RA118.003740

**Published:** 2018-06-29

**Authors:** Sara Rodriguez-Acebes, Silvana Mourón, Juan Méndez

**Affiliations:** From the DNA Replication Group, Molecular Oncology Programme, Spanish National Cancer Research Centre, 3 Melchor Fernández Almagro, 28029 Madrid, Spain

**Keywords:** DNA replication, molecular biology, cell division cycle 7-related protein kinase (Cdc7), DNA polymerase, DNA primase, fork speed, replication origin, replicative stress, stretched DNA fibers

## Abstract

In growing cells, DNA replication precedes mitotic cell division to transmit genetic information to the next generation. The slowing or stalling of DNA replication forks at natural or exogenous obstacles causes “replicative stress” that promotes genomic instability and affects cellular fitness. Replicative stress phenotypes can be characterized at the single-molecule level with DNA combing or stretched DNA fibers, but interpreting the results obtained with these approaches is complicated by the fact that the speed of replication forks is connected to the frequency of origin activation. Primary alterations in fork speed trigger secondary responses in origins, and, conversely, primary alterations in the number of active origins induce compensatory changes in fork speed. Here, by employing interventions that temporally restrict either fork speed or origin firing while still allowing interrogation of the other variable, we report a set of experimental conditions to separate cause and effect in any manipulation that affects DNA replication dynamics. Using HeLa cells and chemical inhibition of origin activity (through a CDC7 kinase inhibitor) and of DNA synthesis (via the DNA polymerase inhibitor aphidicolin), we found that primary effects of replicative stress on velocity of replisomes (fork rate) can be readily distinguished from primary effects on origin firing. Identifying the primary cause of replicative stress in each case as demonstrated here may facilitate the design of methods to counteract replication stress in primary cells or to enhance it in cancer cells to increase their susceptibility to therapies that target DNA repair.

## Introduction

**Figure 1.**
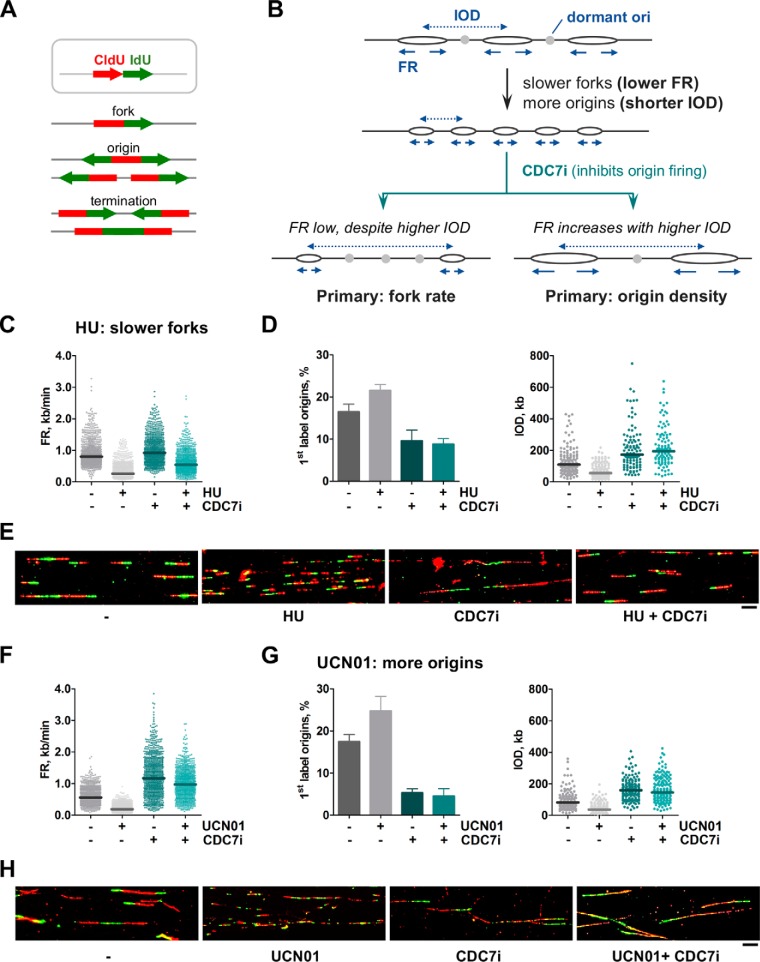
**A CDC7i-based test to separate cause and effect when FR is reduced and origin density is increased.** *A*, DNA replication patterns detected in stretched DNA fibers labeled with CldU and IdU. *B*, schematic of an experimental situation leading to simultaneous reduction in FR (*arrows*) and IOD (*dashed arrows*). The use of CDC7i can determine the primary and secondary effects. See text for details. *C–E*, HeLa cells were treated with HU to reduce FR and IOD. When indicated, CDC7i was added for 12 h. *C*, dot plots show the distribution of FR values (from *left* to *right*, *n* = 896, 935, 876, and 914). *D*, estimation of origin activity calculated as percentage of origin structures (*left panel*; *n* = 1554, 1518, 1509, and 1511 total structures) or IOD values (*right panel*; *n* = 142, 157, 104, and 101). *E*, representative images of DNA fibers under the conditions described. *Scale bar* = 10 μm. *F–H*, HeLa cells were treated with UCN01 to reduce IOD and FR. When indicated, CDC7i was added for 4 h. *F*, distribution of FR values (*n* = 917, 923, 932, and 912). *G*, percentage of origin structures (*left panel*; *n* = 1533, 1545, 1526, and 1571 total structures) and distribution of IOD values (*right panel*; *n* = 152, 153, 152, and 151). *H*, representative images of DNA fibers under the conditions described in *F* and *G. Scale bar* = 10 μm. In dot plots, *horizontal lines* indicate median values. Bar graphs represent mean ± S.D. Data were pooled from three independent experiments.

In proliferating cells, DNA replication precedes mitotic division to allow the transmission of genomic information between generations. The protein machinery responsible for new DNA synthesis is recruited to thousands of replication origins in the G_1_ phase of the cell division cycle. Two replisomes are assembled at each origin that, upon their activation in S phase, move away from each other, establishing bidirectional replication forks. The duration of S phase is determined by the number of active origins and the velocity of replisomes, normally referred to as “fork speed” or “fork rate” (FR).^3^ Both parameters can be analyzed at the single-molecule level using DNA combing or stretched chromatin fibers in cells sequentially labeled with nucleotide analogues CldU and IdU (1, 2). Following cell lysis, labeled DNA molecules are stretched into glass slides, and the incorporation of analogues is visualized by immunofluorescence with separate fluorophores (*e.g.* red for CldU and green for IdU) to identify specific replication structures such as origins, forks, and termination events (Fig. 1*A*). Although FR is directly proportional to the length of labeled tracks and can be quantified using a conversion factor between microscopy image pixels and DNA length (3), the number of active origins can only be estimated indirectly. The average distance between adjacent origins (inter-origin distance, IOD) is frequently used as a proxy for origin density, as it is inversely proportional to the density of active origins. However, accurate IOD estimations require long and stable DNA fibers, and a practical alternative is to quantify the number of origins activated during the CldU pulse (referred to as “first-label origins”) relative to a fixed number of total replication structures, including origins, forks, and termination events (4). Additional parameters can be analyzed in DNA fibers, such as fork stalling and restart of DNA synthesis (5), aberrant origin reactivation (6, 7), and resection of newly synthesized DNA by exonucleases (8). Hence, molecular combing and stretched DNA fibers have become gold standard techniques to examine DNA replication dynamics.

Replication forks are slowed down and occasionally stopped as they encounter special DNA structures, transcription proteins, or DNA lesions introduced by radiation or toxic chemicals. This phenomenon is referred to as “replicative stress” (RS) and is normally counteracted by mechanisms that protect stalled forks and promote the restart of DNA synthesis (9–12). Excessive levels of RS are linked to genomic instability and impinge on many biological processes, including stem cell fitness, senescence, aging, and oncogenic transformation (13–18).

At the molecular level, the interpretation of RS phenotypes is complicated by a marked interdependency between FR and origin activity. Alterations that slow down forks trigger the activation of otherwise “dormant” origins as a compensatory mechanism (19, 20), whereas alterations that primarily increase the number of origins lead to slower forks because the additional replisomes compete for a limited pool of dNTPs and possibly other factors. Similarly, an increase in the pool of dNTPs accelerates fork progression and reduces the likelihood of activation of neighboring origins, whereas other manipulations that limit origin activation yield faster forks because of higher dNTP availability (21–24). In all cases, origin activity and fork speed influence each other, making it difficult to ascertain the primary cause of stress. In this study, we have devised and tested experimental conditions to address this “chicken and egg” causality problem between fork speed and origin activation, based on interventions that temporally restrict one of the two parameters while still allowing interrogation of the other.

## Results and discussion

We first examined the two possible cases that lead to a concomitant slowdown of forks (*i.e.* lower FR) and increase in origin density (*i.e.* shorter IOD) in HeLa cells (Fig. 1*B*). Experimental conditions were chosen for which the primary effect could be inferred beforehand. For instance, hydroxyurea (HU) inhibits ribonucleotide reductase and reduces the pool of dNTPs, causing a decrease in FR that subsequently activates dormant origins (the unlikely alternative would be that HU promotes origin firing, slowing forks as a consequence). To confirm that the former interpretation is correct, the effect of HU on FR and origin activity was tested in the absence or presence of a CDC7 kinase inhibitor (CDC7i). CDC7 kinase activates the MCM helicase and is necessary for origin firing (25, 26); its depletion with RNAi has been used to establish the link between replication initiation and fork progression (4). In our study, the concentration of CDC7i was adjusted to reduce origin activity without inhibiting DNA synthesis altogether; addition of 60 μm CDC7i for 4–12 h was sufficient to reduce MCM2 phosphorylation (Fig. S1*A*) and restrict origin activation, as indicated by the lower percentage of first-label origins and increased inter-origin distances (Fig. S1, *B–D*). At this CDC7i concentration, the global effects on DNA synthesis and cell cycle distribution were modest (Fig. S1, *E* and *F*).

Because HU affects fork progression independently of origin activity, the reduction in FR should be observed regardless of the presence of CDC7i (Fig. 1*B*, *left branch*). If, on the other hand, the effect of HU depended on the activation of extra origins, then it would be corrected by CDC7i (Fig. 1*B*, *right branch*). The decrease in FR and concomitant activation of new origins were readily detected after HU treatment (Fig. 1, *C* and *D*; compare the *two leftmost sets of data* in each graph). As anticipated, when HU was combined with CDC7i, FR remained low despite a strong block in origin activity (Fig. 1, *C–E*). The slight increase in FR relative to the HU-only conditions can be explained as a secondary effect of CDC7i itself following origin repression (Fig. 1*C*, compare *first* and *third dot plots*; see also Supp. Fig. 1*C*).

The opposite situation, *i.e.* a primary increase in origin activity, was tested using UCN01, an inhibitor of the checkpoint kinase CHK1, which promotes promiscuous origin activation (20, 27, 28). The positive effect of UCN01 on origin activation was confirmed by the increased percentage of first-label origins and reduced IOD values, as was the concomitant reduction in FR (Fig. 1, *F* and *G*, the *two leftmost sets of data* in each graph). In this case, however, upon addition of CDC7i, the negative effect of UCN01 on FR was attenuated and reached similar levels as treatment with CDC7i alone (Fig. 1, *F–H*). This result confirms that the change in FR is a secondary response that follows the primary influence of UCN01 on origin activation.

**Figure 2.**
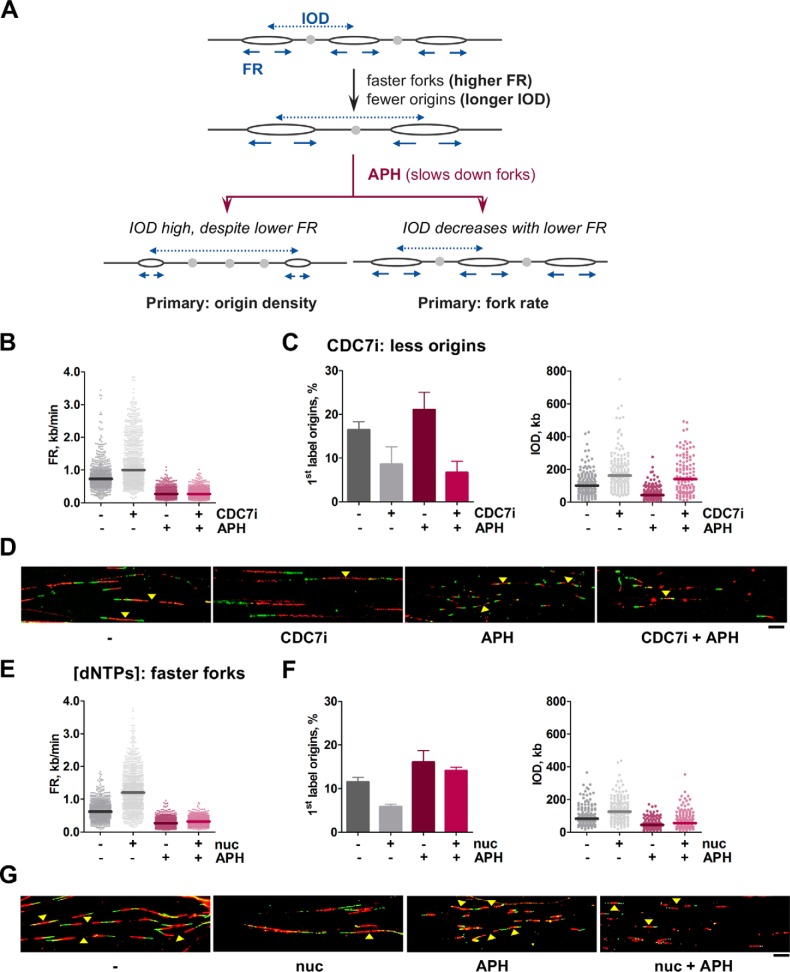
**A test to separate cause and effect when FR is increased and origin density is reduced.** *A*, schematic of an experimental situation leading to simultaneous increase in FR (*arrows*) and IOD (*dashed arrows*). The use of APH allows discrimination of the primary and secondary effects. See text for details. *B–D*, HeLa cells were treated with CDC7i for 4 h to increase IOD and FR. When indicated, APH was added for 2 h. *B*, dot plots showing distribution of FR values (from *left* to *right*, *n* = 889, 881, 905, and 927). *C*, estimation of origin activity by the percentage of first-label origin structures (*left panel*; *n* = 1539, 1529, 1529, and 1527) and distribution of IOD values (*right panel*; *n* = 153, 147, 157, and 114). *D*, representative pictures of DNA fibers in HeLa cells treated as described. *Yellow arrowheads* point to origins. *Scale bar* = 10 μm. *E–G*, cell medium was supplemented with extra nucleosides for 4 h to increase FR and IOD. When indicated, APH was added for 2 h. *E*, distribution of FR values (from *left* to *right*, *n* = 929, 921, 915, and 925). *F*, origin activity estimated as percentage of origin structures (*left panel*; *n* = 1549, 1497, 1542, and 1529 total structures) and distribution of IOD values (*right panel*; *n* = 153, 152, 157, and 156). *G*, representative pictures of DNA fibers in HeLa cells treated as described in *E* and *F. Yellow arrowheads* point to origins. *Scale bar*, 10 μm. In dot plots, *horizontal lines* indicate median values. Bar graphs represent mean ± S.D. Data were pooled from three independent experiments.

Although RS commonly refers to the slowdown of forks, there are also experimental scenarios in which forks become faster while origin activity decreases (*i.e.* longer IOD; Fig. 2*A*). In these cases, in which the use of CDC7i would be redundant with the defect in origin activity, another intervention was designed based on the DNA polymerase inhibitor aphidicolin (APH) to temporarily reduce FR. The rationale is parallel to the situation described before: if the observed reduction in origin density is the primary effect and does not depend on fork speed, then it should be resilient to APH interference. On the contrary, if it is a secondary response to faster forks, then it would be corrected by APH (Fig. 2*A*, compare the *left* and *right branches*). Optimization experiments indicated that a short treatment with 5 μm APH was sufficient to slow down forks (Fig. 2, *A–C*) without abolishing DNA synthesis or significantly altering the cell cycle (Fig. S2, *D* and *E*).

To validate the APH test, conditions were found that exerted a primary influence either on origin density or in FR. For the first case, cells were treated with CDC7i, which restricts origin activation and elicits a secondary increase in FR. As anticipated, origin density was reduced by CDC7i treatment and remained low even when FR was markedly reduced by APH (Fig. 2, *B–D*). The complementary case was tested by supplementing the cell medium with extra nucleosides to promote faster fork progression, causing a secondary decrease in origin activation (21, 24, 29). In this case, the effect of nucleosides on origin activity strictly depended on FR, as it was largely corrected by the addition of APH (Fig. 2, *E–G*). These results confirm that the primary effect of nucleoside addition was to accelerate FR.

**Figure 3.**
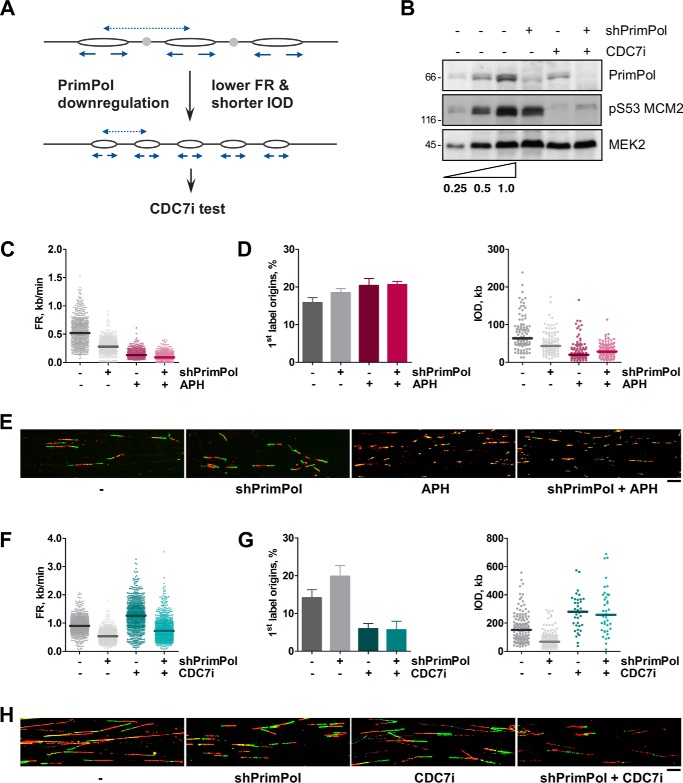
**The CDC7i test determines that low FR is the primary effect of PrimPol down-regulation.** *A*, schematic of the simultaneous reduction in FR and IOD (higher origin density) caused by PrimPol down-regulation in HeLa cells (data shown in *C–H*). The CDC7i test can be applied. *B*, immunoblots showing the down-regulation of PrimPol and the effect of CDC7i on MCM2 phosphorylation at Ser-53. MEK2 levels are shown as a loading control. The *first two lanes* show serial 2-fold dilutions of the control condition (*third lane*) for comparison purposes. *C–E*, the result of applying the APH test. HeLa cells were treated with doxycycline for 4 days to induce shPrimPol, and 5 μm APH was added for 2 h when indicated. *C*, distribution of FR values (from *left* to *right*; *n* = 617, 615, 617, and 627). *D*, origin activity as determined by percentage of first-label origin structures (*left panel*; *n* = 1037, 1037, 1013, and 1044 total structures) and distribution of IOD values (*right panel*; *n* = 102, 107, 105, and 103). *E*, representative images of DNA fibers in HeLa cells treated as in *C* and *D. Scale bar* = 10 μm. *F–H*, HeLa cells were treated with doxycycline for 4 days to induce shPrimPol, and 60 μm CDC7i was added for 12 h when indicated. *F*, distribution of FR values (from *left* to *right*, *n* = 934, 979, 901, and 914). *G*, origin activity determined by percentage of first-label origin structures (*left panel*; *n* = 1514, 1588, 1641, and 1525 total structures) and distribution of IOD values (*right panel*; *n* = 141, 157, 37, and 39). *H*, representative images of DNA fibers in HeLa cells treated as indicated in *F* and *G. Scale bar* = 10 μm. In the dot plots, median values are indicated by *horizontal lines*. Bar graphs represent mean ± S.D. In *C–E,* data were pooled from two independent experiments. In *F–H,* data were pooled from three independent experiments.

Both the CDC7i- and APH-based “causality tests” work through a temporal disruption in the link between fork rate and origin activity and allow identification of the primary influence of different chemical or genetic manipulations on DNA replication. As proof of concept, we have applied them to a specific problem arising from our recent research on PrimPol, a protein with primase and polymerase activities that participates in replicative tolerance to damaged DNA (5, 30–32). PrimPol down-regulation in HeLa cells simultaneously reduced FR and IOD (slower forks, higher origin density), which could be attributed to a role of PrimPol either at forks or origins (5) (Fig. 3). According to the examples described in Figs. 1 and 2, the test based on CDC7i should be applied, whereas the test based on APH would exacerbate the FR phenotype. To demonstrate that this is the case, both tests were applied to HeLa cells following PrimPol down-regulation (Fig. 3*B*). As anticipated, APH acutely decreased FR and increased origin activity (Fig. 3, *C–E*), enhancing the alterations induced by the loss of PrimPol. In this context, APH does not contribute to discriminate whether PrimPol primarily affects FR or origin usage. In turn, the use of CDC7i revealed that the reduction in FR caused by PrimPol down-regulation was maintained even when extra origins could not be activated (Fig. 3, *F–H*). This result indicates that the loss of PrimPol affects fork progression directly, in line with other experiments that support a role for PrimPol in facilitating fork progression through UV-damaged DNA (5) and G quadruplex structures in S phase (33).

The CDC7i- and APH-based tests described here are rapid and straightforward and may be applied to a large variety of experimental conditions. However, alternative tests may be designed that follow the same underlying principle, *i.e.* to counteract one of the two parameters while allowing for the interrogation of the other. For instance, in the case of simultaneous reduction of FR and IOD, CDK inhibitors could be considered instead of CDC7i when the conditions of the experiment advise against the inhibition of DDK kinase. In the case of a simultaneous increase in FR and IOD, hydroxyurea could substitute for APH when inhibition of DNA polymerases is to be avoided for any reason. We encourage researchers to use these types of tests to determine the primary effect of any chemical or genetic manipulation on the dynamics of DNA replication. Learning the precise mechanism underlying RS in each experimental setting will separate events that actually delay fork progression from those that rather affect origin activity; this information could then be applied to design strategies to counteract RS in stem cells, enhancing their long-term functionality (15, 16). Another promising prospect would be to enhance RS in cancer cells to increase their sensitivity to therapies targeting DNA repair pathways or the checkpoint proteins ATR and Chk1 (34–36). Finally, we hope that these tests may contribute to further understanding of the impact of oncogene activation on DNA replication and chromosome fragility (37–40).

## Experimental procedures

### Cell culture and manipulations

HeLa cells were grown in Dulbecco's modified Eagle's medium supplemented with 10% fetal bovine serum plus penicillin–streptomycin. To down-regulate PrimPol, a stable HeLa-shPrimPol cell line generated in our laboratory was used (5). Short hairpin RNA expression was induced with 1 μg/ml doxycycline (Sigma) for 96 h. Hydroxyurea, PHA767491 (CDC7i), APH, and UCN01 were obtained from Sigma. Embryomax^TM^ nucleosides were obtained from Millipore. Unless otherwise indicated, the following drug concentrations and incubation times were used: 200 μm HU (2 h), 60 μm CDC7i (12 h), 10 nm UCN01 (5 h), 5 μm APH (2 h), and 150 μm nucleosides (4 h).

### Single-molecule analysis of DNA replication in stretched DNA fibers

Cells were pulse-labeled (20 min) with 50 μm CldU followed by 250 μm IdU (20 min) prior to cell harvesting and lysis in 0.2 m Tris (pH 7.4), 50 mm EDTA, and 0.5% SDS. Stretched DNA fibers were prepared as described previously (5). For immunodetection of labeled tracks, fibers were incubated with anti-CldU (rat monoclonal anti-BrdU, Abcam, AB6326), anti-IdU (mouse monoclonal anti-BrdU, BD Biosciences, 347580), and anti-single-stranded DNA (Millipore, MAB3034) for 1 h at room temperature in a humidity chamber. Alexa Fluor-conjugated secondary antibodies (Invitrogen/Molecular Probes, A-11007, A-21121, and A-21241) were used for 30 min at room temperature. Images were obtained with a DM6000 B Leica microscope with a HCX PL APO ×40 0.75 NA objective. The conversion factor used was 1 μm = 2.59 kb (3). Signals were measured and quantified using ImageJ software (41). For FR, 250–350 forks (red–green tracks) were measured. For IOD, 15–50 measurements between two adjacent origins on intact fibers were taken. For origin firing, origins labeled during the first pulse (green–red–green structures) were quantified as percentage of all structures containing red (>500 total structures scored in each case).

### Immunoblots and antibodies

Whole-cell extracts were prepared by suspension of cells in Laemmli buffer followed by sonication (3 × 15 s in a Branson digital sonifier set at 15% amplitude). SDS-PAGE, protein transfer to nitrocellulose membranes and immunoblots were performed using standard protocols. Anti-Mcm2 and anti-PrimPol have been described previously (5, 39). The following commercial antibodies were used: anti-MCM2-pS53 (Abcam, AB70367), anti-MCM2-pS40 (Abcam, AB133243), and anti-MEK2 (BD Biosciences/Pharmingen, 610236).

### Flow cytometry detection of IdU incorporation and DNA content

Cells were pulse-labeled with 250 μm IdU for 20 min, collected, and fixed in 70% ethanol. DNA was denatured by incubation in 2 n HCl for 20 min, washed, and incubated with FITC-conjugated anti-BrdU (BD Biosciences/Pharmingen, 556028) for 60 min at 37 °C. To monitor DNA content, cells were stained with 50 μg/ml propidium iodide (Sigma) in the presence of 10 μg/ml RNase A (Qiagen). Flow cytometry was performed in a FACSCanto II cytometer (BD Biosciences) and analyzed with FlowJo 10 (Tree Star).

## Author contributions

S. R.-A. and J. M. conceptualization; S. R.-A. and J. M. formal analysis; S. R.-A. and S. M. investigation; S. R.-A. and J. M. writing-original draft; S. R.-A. writing-review and editing; J. M. supervision; J. M. funding acquisition.

## Supplementary Material

Supporting Information

## Footnotes

3 The abbreviations used are:

FR: fork rate
IOD: inter-origin distance
RS: replicative stress
HU: hydroxyurea
CDC7i: CDC7 inhibitor
APH: aphidicolin
BrdU: bromodeoxyuridine
CldU: chlorodeoxyuridine
IdU: iododeoxyuridine
MCM: mini-chromosome maintenance.
