# Supplementary material for: Uncoupling fork speed and origin activity to identify the primary cause of replicative stress phenotypes
Source: J Biol Chem. 2018 Jun 29;293(33):12855–61. doi: 10.1074/jbc.RA118.003740 (PMC6102153; doi:10.1074/jbc.RA118.003740)
Supplement: Supporting Information [file supp_293_33_12855__index.html]

Uncoupling fork speed and origin activity to identify the primary cause of replicative stress phenotypes — Cause and effect in replicative stress — Uncoupling fork speed and origin activity to identify the primary cause of replicative stress phenotypes — Cause and effect in replicative stress — Supporting Information 

# Uncoupling fork speed and origin activity to identify the primary cause of replicative stress phenotypes

## Supporting Information

- Supplementary Information - Two supplementary Figures with legends
