# Supplementary material for: Uncoupling fork speed and origin activity to identify the primary cause of replicative stress phenotypes
Source: J Biol Chem. 2018 Jun 29;293(33):12855–61. doi: 10.1074/jbc.RA118.003740 (PMC6102153; doi:10.1074/jbc.RA118.003740)
Supplement: Supporting Information [file supp_RA118.003740_137814_1_supp_159254_pcvxcz.pdf]

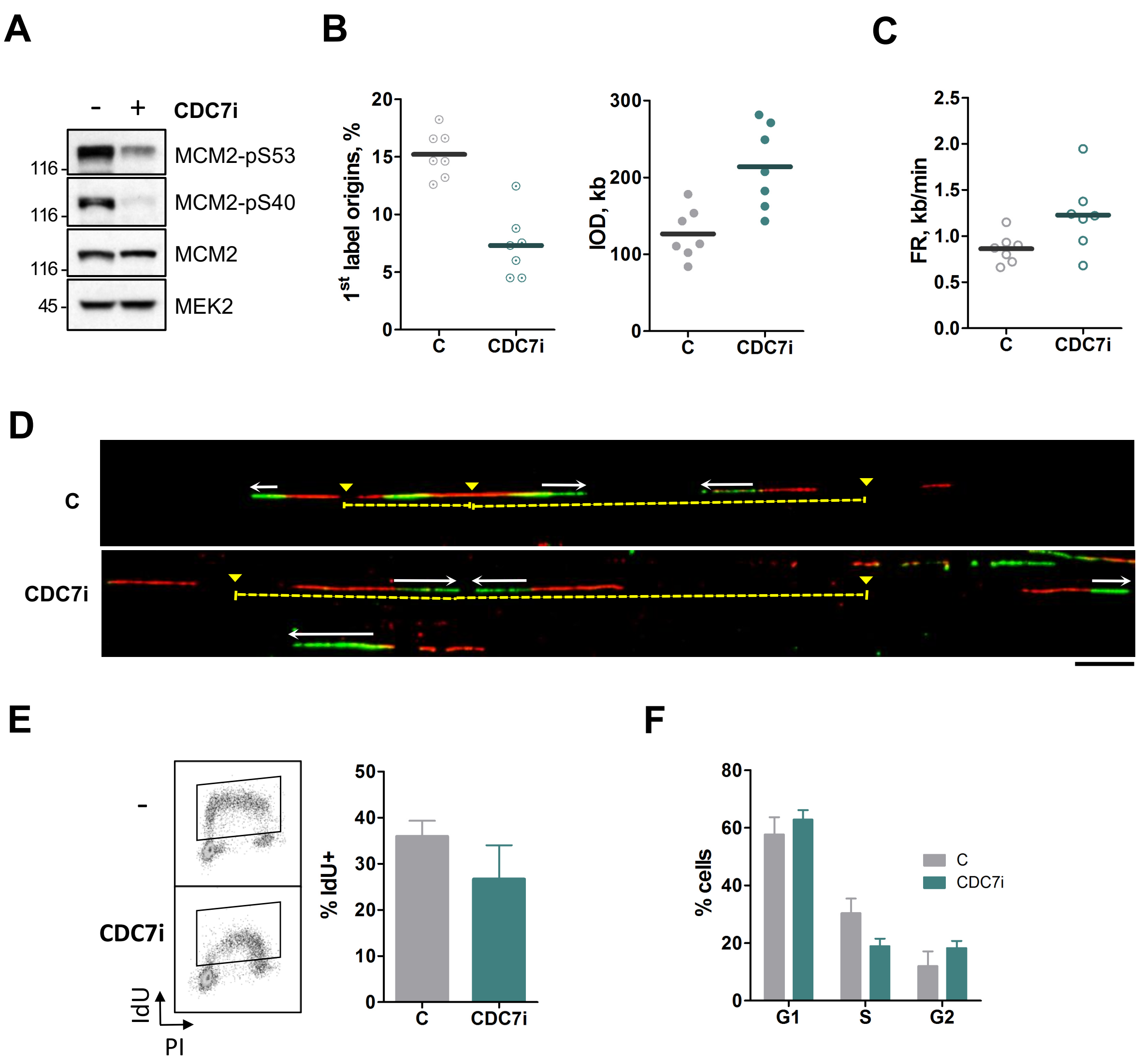

Figure S1. CDC7i restricts origin activity. HeLa cells were grown in the presence of 60 µM CDC7i for 12 h. *A,* immunoblot detection of the indicated proteins. MEK2 is shown as loading control. *B.* Median percentage of first-label origin structures relative to the total number of red tracks (left) and median IOD values (right) in seven independent experiments. Dots indicate the median value of each experiment; horizontal bars indicate the average of the medians. 15-50 IOD values were taken in each replicate experiment. *C,* median FR values corresponding to the same experiments; in each experiment, 250-350 FR values were taken. *D,* representative images of labeled DNA fibers. White arrows, forks; yellow arrowheads, origins; dashed yellow lines, IOD. Bar, 10 µm. *E*, flow cytometry analysis of IdU incorporation. One representative experiment is shown (left). Histogram represents the quantification of seven experiments (mean+S.D.). *F,* Distribution of the cell population in the different cell cycle phases, calculated from flow cytometry analyses of DNA content. Histogram shows the quantification of seven independent experiments (mean+S.D.).

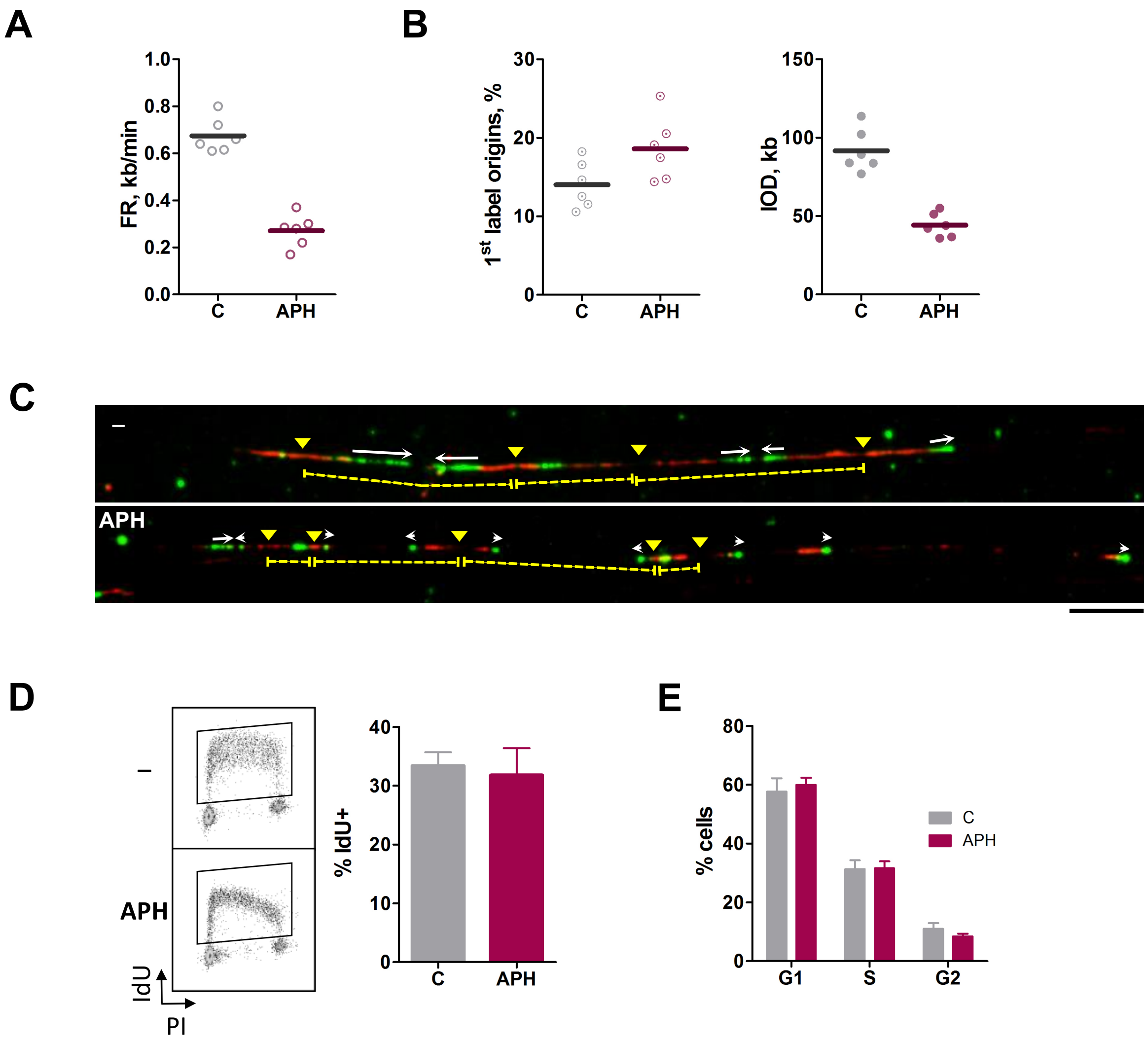

Figure S2. APH reduces fork progression. HeLa cells were grown in the absence or presence of 5 µM aphidicolin (APH) for 2 h. *A*, median FR values in six independent experiments; in each experiment, 250-350 FR values were taken. *B*. Median percentage of first-label origin structures relative to the total number of red tracks (left) and median IOD values (right) corresponding to the same experiments. Dots indicate the median value of each experiment; horizontal bars indicate the average of the medians. 15-50 IOD values were taken in each replicate experiment. *C,* representative images of labeled DNA fibers. White arrows, forks; yellow arrowheads, origins; dashed yellow lines, IOD. Bar, 10 µm. *D*, flow cytometry analysis of IdU incorporation. One representative experiment is shown (left). Histogram represents the quantification of six experiments (mean+S.D.). *E,* Distribution of the cell population in the different cell cycle phases, calculated from flow cytometry analyses of DNA content. Histogram shows the quantification of six independent experiments (mean+S.D.).
